# Highly Conserved but Activation‐Dependent CCR5, CCR6, and CXCR4 Chemokine Receptor Expression and Trophoblast‐Mediated Cytokine Profile of Peripheral Gamma‐Delta T Cells During Early Human Pregnancy

**DOI:** 10.1155/jimr/7743945

**Published:** 2026-10-23

**Authors:** Banan Alrawashdeh, Jasper Nörenberg, Pál Jáksó, Barbara Forró, Mátyás Meggyes, Ákos Várnagy, Aliz Barakonyi

**Affiliations:** ^1^ Department of Medical Microbiology Clinical Center University of Pécs Medical School Pécs Hungary pte.hu; ^2^ Department of Obstetrics and Gynaecology Clinical Center University of Pécs Medical School Pécs Hungary pte.hu; ^3^ National Laboratory on Human Reproduction University of Pécs Pécs Hungary pte.hu; ^4^ Department of Pathology Clinical Center University of Pécs Medical School Pécs Hungary pte.hu

**Keywords:** chemokine receptors, conventional γδT cells, cytokines, early human pregnancy, NK-like γδT cells, peripheral blood, trophoblast cells

## Abstract

Pregnancy requires balanced maternal immune regulation for healthy fetal development, in which γδT cells contribute at the feto‐maternal interface; however, mechanisms guiding decidual γδT‐cell accumulation remain unclear. We analyzed chemokine receptor (ChR) expression (C‐C motif ChR [CCR]5, CCR6, and C‐X‐C motif ChR [CXCR]4) and cytokine profiles of peripheral γδT cells from first‐trimester pregnant and nonpregnant women by multicolor flow cytometry under baseline and antigen‐independent activation, while cytokine modulation was assessed in isolated γδT cells co‐cultured with trophoblasts using cytometric bead array (CBA). While the frequency of ChR‐expressing γδTCR^+^ cells was largely preserved, CCR6 and CXCR4 expression intensities were slightly elevated during pregnancy. Although single ChR expression did not differ between conventional and natural killer (NK)‐like γδT cell subsets, the proportions of some ChR co‐expressing populations were slightly but significantly higher among NK‐like γδT cells. Within the observed co‐expression patterns, CCR5/CCR6 was the most frequent combination on γδT cell subsets. Furthermore, different stimulation models differently shaped ChR expression on conventional γδT cells, with physiological activation maintaining CCR5^+^ dominance alongside elevated CXCR4^+^ cell ratios, whereas PMA/Ionomycin stimulation was characterized by a predominance of CXCR4^+^ cells and a low proportion of CCR5^+^ cells. In terms of cytokine production γδT cells exhibited a broad Th1‐like cytokine profile dominated by IL‐2, TNF‐α, and IFN‐γ, with modest reductions in TNF‐α and IFN‐γ during pregnancy. Trophoblast contact further attenuated this response by inhibiting IL‐2, TNF‐α, and IL‐17A release, while markedly enhancing IL‐6 production. Our findings suggest that γδT cell subsets slightly differ in ChR co‐expression patterns, and ChRs are differentially regulated by distinct activation processes. The modest differences observed during first‐trimester pregnancy indicate a conserved migratory capacity, with pregnancy‐specific migratory phenotypes likely shaped at the materno‐fetal interface. Additionally, γδT cells exhibited an inflammatory cytokine milieu that was modulated upon trophoblast contact, which may contribute to trophoblast‐mediated immune adaptations during human pregnancy.

## 1. Introduction

The coexistence of two genetically and immunologically distinct individuals within a single body during pregnancy raises fundamental challenges to the classical concepts of transplantation and tumor physiology. In this context, the fetus inherits and expresses paternal antigens, and fetal trophoblast cells come into direct contact with the maternal immune system at the materno‐fetal interface [1]. Rather than mounting an immune response against these foreign antigens, maternal immune regulation aims to balance effective protection against pathogens while maintaining tolerance to the semi‐allogeneic fetus [2]. Accumulating evidence suggests that the materno‐fetal interface is immunologically active rather than inert and that a successful pregnancy requires a flexible, robust, and responsive maternal immune adaptation [3]. Among the critical parameters that play a key role in establishing immune tolerance to fetal antigens are maternal decidual lymphocytes, with the vast majority belonging to the innate immune system [4], including natural killer (NK) cells, NK‐like T cells, and γδT cells.

While the majority of mature T lymphocytes are TCRαβ^+^, TCRγδ^+^ cells constitute only 1%–10% of peripheral blood T cells [5]; however, they are enriched in several epithelial and mucosal barrier tissues, such as the intestine and female reproductive tract [6]. Decidual γδT cells have been implicated in supporting trophoblast proliferation and invasion, placental development, and the maintenance of a balanced immune environment, partly through the production of regulatory cytokines [7, 8]. Previous studies showed that decidual lymphocytes increase in number at the materno‐fetal interface during early pregnancy, representing a highly specialized immune cell population with distinct phenotypic and functional characteristics [9]. Among these, decidual NK cells are the dominant cell population, accounting for approximately 70%, followed by macrophages (20%) and T cells (10%) [10]. Despite considerable progress in characterizing the phenotypes and functions of decidual immune cells (DICs), their precise origin—whether through local differentiation from uterine progenitor cells or recruitment from peripheral tissues, or both—remains one of the most debated questions in reproductive immunology. While most of the current knowledge is derived from studies on NK cells, little is known about how other lymphocyte populations, such as γδT cells, become established in the decidua. Given the pivotal role of the interactions between chemokines and chemokine receptors (ChRs) in leukocyte trafficking [11], it is likely that the recruitment of DICs is mediated, at least in part, by the expression and secretion of chemokines at the feto‐maternal interface. Indeed, chemokine signaling has been shown to play critical roles in embryo implantation and the regulation of trophoblast invasion [12]. Since decidual CD56^bright^ NK cells represent the predominant immune cell subset, chemokines involved in their recruitment have received considerable attention [13, 14]. Accordingly, decidual and trophoblast cells were found to produce NK‐attracting chemokines, and evidence suggests that decidual CD56^bright^ cells are recruited through the C‐X‐C motif ChR 3 (CXCR3)–C‐X‐C motif chemokine ligand CXCL10 and CXCR4–CXCL12 axes, with CXCL12 also known as stromal cell‐derived factor 1 (SDF‐1), secreted by trophoblast cells [15, 16]. By contrast, the origin of decidual γδT cells has received far less attention, despite the higher prevalence of these cells among decidual CD3^+^ cells compared to peripheral blood [7]. A previous study demonstrated that CXCL16 produced by trophoblast cells plays a role in γδT cell migration by binding to CXCR6; however, whether other ChRs also contribute to this process remains to be further investigated [17]. The C‐C motif ChR 5 (CCR5) is characteristically associated with Th1‐type immune responses and is preferentially expressed on peripheral blood Vγ9Vδ2 cells [18, 19]. CCR5 is one of the receptors for C‐C motif chemokine ligand 5 (CCL5, also known as regulated upon activation, normal t‐cell expressed and secreted [RANTES]) and plays an important role in inflammatory responses [20]. It has been demonstrated that CCL5 is produced by human decidual tissue, acting as a chemoattractant for invading trophoblast cells and the blastocyst, suggesting a potential role in implantation, but whether the CCR5–CCL5 axis contributes to decidua‐specific recruitment has not been established yet [21]. Another ChR that is also expressed on γδT cells, CCR6, the sole receptor for CCL20 (macrophage inflammatory protein‐3α [MIP‐3α]) [22]. Human studies showed that the CCR6–CCL20 axis plays an important role in guiding CD4^+^/Forkhead box P3 (FOXP3)^+^ Treg migration toward the feto‐maternal interface. Moreover, the same study reported lower peripheral blood and decidual CCR6–CCL20 expression levels in patients with unexplained recurrent miscarriage during the early embryonic period, suggesting its importance in maintaining an appropriate immunoregulatory environment [23]. Furthermore, porcine models showed that CCR6–CCL20 signaling promotes the proliferation of endometrial epithelial cells, potentially preparing the endometrium for implantation and placentation [24]. Taken together, interactions involving CXCR4/CXCL12, CCR5/CCL5, and CCR6/CCL20 appear to be important for maintaining a healthy pregnancy. However, data regarding the expression patterns of CCR5, CCR6, and CXCR4 on γδT cells and whether they are modulated by pregnancy status are still lacking. Given the established roles of these ChRs in leukocyte trafficking and pregnancy‐related processes, their combined analysis in γδT cells may provide a more comprehensive characterization of γδT‐cell migratory potential and clarify whether they are differentially expressed on other innate‐like immune cells during pregnancy.

In this study, we analyzed distinct immune cell populations that share key functional properties, including rapid effector function and the expression of NK‐associated markers, allowing them to be grouped together as a common functional subset. We refer to this group as “Natural and Innate‐Like Lymphocytes” (NILLs), comprising four flow cytometrically defined populations (CD3^‒^ conventional NK cells, CD56^‒^ conventional γδT cells, CD56^+^ NK‐like γδT cells, and CD56^+^ NK‐like non‐γδT cells). The latter two subpopulations can be grouped under the NK‐like T cell subset, as they both express CD3 and CD56 but differ in their TCR. Although CD56^+^ NK‐like γδT cells have been reported to increase in peripheral blood during the second half of human gestation [25], they remain poorly characterized during pregnancy, making their inclusion in our analysis particularly relevant. We focused on these four NILL populations to characterize their migratory potential during the first‐trimester pregnancy. ChR expression profiles and intensity (CXCR4, CCR5, and CCR6) were assessed in peripheral blood samples obtained from healthy nonpregnant women and women during the first trimester of pregnancy. We further determined the expression levels of ChRs and the associated differences following antigen‐independent activation. Among the NILL subsets, conventional γδT cells were examined in more detail, as this population is not fully characterized in the context of pregnancy; thus, we evaluated whether exposure to trophoblast cells alters their cytokine production profile.

## 2. Materials and Methods

### 2.1. Human Samples

Peripheral blood samples were collected from healthy, nonpregnant female volunteers (*n* = 36) at the Department of Medical Microbiology, University of Pécs, Medical School, Hungary. Samples from healthy women in the first trimester of pregnancy (gestational weeks 7–13, *n* = 31) were obtained at the Department of Obstetrics and Gynecology, University of Pécs, Medical School, Hungary. The demographic and obstetric characteristics of the study participants are summarized in Table 1. All participants were free of chronic diseases, autoimmune conditions, and reproductive disorders such as endometriosis or polyendocrine metabolic ovarian syndrome, formerly known as polycystic ovary syndrome. Individuals with hypothyroidism receiving hormone replacement therapy were also excluded.

**Table 1:** Demographic and obstetric characteristics of pregnant participants and nonpregnant controls.

Characteristics	Pregnant group (*n* = 31)	Nonpregnant controls (*n* = 36)	*p*‐Value
Demographic characteristics			
Age (years)	28.87 ± 5.99	29.13 ± 7.22	0.854 (NS)
Obstetric characteristics			
Gestational age (weeks)	11.50 ± 1.50	N/A	—
Previous pregnancy history			
≥1 previous pregnancy *n* (%)	13 (48.1%)	13 (40.6%)	0.607 (NS)

*Note*: Data are presented as mean ± standard deviation (SD) or number (percentage), as appropriate. Previous pregnancy history was available for 27 of 31 pregnant participants and 32 of 36 nonpregnant controls. Information on the time elapsed since the most recent pregnancy was not collected. Between‐group comparisons were performed using Student’s *t*‐test or Fisher’s exact test, as appropriate.

Abbreviations: N/A, not applicable; NS, not significant.

### 2.2. Isolation of Peripheral Blood Mononuclear Cells (PBMCs)

Peripheral blood (10 mL) was collected from each donor into sodium‐heparin‐containing tubes and immediately diluted with 20 mL of phosphate‐buffered saline (PBS) (Biosera). The diluted blood was gently layered onto 10 mL Ficoll‐Paque solution (GE Healthcare), and PBMCs were isolated by density gradient centrifugation at 670 × *g* for 20 min without brake. Following centrifugation, the PBMC layer was collected and washed twice with PBS by centrifugation at 400 × *g* for 7 min to remove residual Ficoll. After the final wash, the cell pellet was resuspended in 2–5 mL PBS, depending on pellet size. Cells were then stained with Türk’s solution, and lymphocytes were manually counted using a Bürker counting chamber under a light microscope.

### 2.3. Magnetic Isolation of Peripheral Blood γδT Cells

γδT cells were isolated from peripheral blood PBMCs by negative selection using the “TCRγδ^+^ T Cell Isolation Kit” (Miltenyi Biotec). The kit contains a primary labeling reagent composed of biotin‐conjugated monoclonal anti‐human antibodies targeting antigens not expressed on TCRγδ^+^ cells, as well as anti‐biotin magnetic MicroBeads. Accordingly, non‐TCRγδ^+^ cells (including αβT cells, NK cells, B cells, dendritic cells, granulocytes, monocytes, stem cells, and erythroid cells) were labeled and retained in the magnetic column, while untouched γδT cells passed through. PBS, supplemented with 0.5% Fetal Bovine Serum (FBS) (Biosera), was used as a buffer throughout the procedure. All labeling and washing steps were performed at 4–8°C. Briefly, the PBMC suspension was passed through a 30‐μm nylon filter to obtain a single‐cell suspension and centrifuged at 300 × *g* for 10 min. For up to 1 × 10^7^ cells, the pellet was resuspended in 80 μL buffer, mixed with 20 μL biotin‐antibody cocktail, and incubated for 10 min at 4–8°C. Cells were then washed with 1–2 mL buffer by centrifugation at 300 × *g* for 10 min, resuspended in 80 μL buffer, mixed with 20 μL anti‐biotin MicroBeads, and incubated for 15 min at 4–8°C. After a second wash under the same conditions, the cell pellet was resuspended in 500 μL buffer and applied to an LS Column (Miltenyi Biotec) pre‐rinsed with 3 mL buffer. The column was subsequently washed three times with 3 mL buffer, and the unlabeled flow‐through containing the enriched γδT‐cell population was collected and centrifuged at 300 × *g* for 10 min. Reagent volumes were scaled proportionally when more than 1 × 10^7^ cells were processed. Thereafter, the purity of the enriched γδT‐cell population was assessed by flow cytometry and found to be 90.78% ± 2.42 (mean ± SD). Only samples containing >90% viable TCRγδ^+^ T cells were used for subsequent co‐culture experiments.

### 2.4. Handling and Activation of PBMCs Prior to Flow Cytometry

Following PBMC isolation and counting, 1 × 10^6^ freshly isolated cells were incubated in 1 mL PBS supplemented with 10% FBS at 4°C for 4 h before antibody staining to preserve ChR surface expression levels [26], hereafter referred to as the baseline group. Another fraction of freshly isolated PBMCs was stimulated under antigen‐independent conditions before flow cytometry labeling to evaluate the potential ChR surface expression patterns under in vitro stimulation. Following cell counting, 1 × 10^6^ cells were transferred into tubes and washed with PBS by centrifugation at 400 × *g* for 7 min. Cell pellets were then resuspended in 1 mL PBS supplemented with 10% FBS, Ionomycin (1 µg/mL) (Sigma–Aldrich), and phorbol myristate acetate (PMA; 25 ng/mL) (Sigma–Aldrich) and incubated for 4 h at 37°C in a humidified atmosphere containing 5% CO_2_. The applied PMA and Ionomycin concentrations were chosen based on previously published protocols and our earlier study, where this approach provided consistent and effective activation of γδT cells [27]. Such activation is also known to influence ChR expression patterns [28].

### 2.5. Flow Cytometry Analysis

1 × 10^6^ cells from each pretreatment condition were washed with PBS by centrifugation at 400 × *g* for 7 min and subsequently stained using an 8‐color flow cytometry panel by simultaneous incubation with eight distinct fluorochrome‐conjugated monoclonal antibodies (mAbs) for 25 min in the dark at room temperature (Table 2). Based on the manufacturers’ recommendations, antibody concentrations were optimized by titration before use. The cells were then washed with PBS by centrifugation at 400 × *g* for 7 min, resuspended and fixed in 500 μL PBS containing 4% paraformaldehyde (PFA), and stored in the dark until flow cytometric analysis the following day. Measurements were performed using a Navios flow cytometer (Beckman Coulter). Compensation matrices were calculated by FlowJo v9.8.5 using BD CompBeads, and further analysis was performed with FCS Express v3 software. Fluorescence minus one (FMO) controls were used to define marker‐specific positivity thresholds. For the assessment of receptor expression, mean fluorescence intensity (MFI) and the proportion of ChR‐positive cells were used as complementary measures of receptor expression, as appropriate for the respective analyses.

**Table 2:** Monoclonal antibodies used for flow cytometric staining of peripheral NILL subsets.

Antibody (target)	Fluorochrome	Isotype	Clone	Manufacturer
Anti‐human‐TCRγδ	Brilliant Violet 421	Mouse IgG1	B1	Sony^a^
Anti‐human‐CD45	Alexa Fluor 700	Mouse IgG1	2D1	Sony^a^
Anti‐human‐CD3	PerCP/Cy5.5	Mouse IgG2a	OKT3	Sony^a^
Anti‐human‐CD56	Brilliant Violet 510	Mouse IgG1	HCD56	Sony^a^
Anti‐human‐CCR5	PE	Rat IgG2b	J418F1	Sony^a^
Anti‐human‐CCR6	APC	Mouse IgG2b	G034E3	Sony^a^
Anti‐human‐CXCR4	PE‐Vio770	Mouse IgG2a	12G5	Miltenyi^b^
Anti‐human‐CD69	FITC	Mouse IgG1	FN50	Sony^a^

*Note*: The table lists anti‐human target antigens, conjugated fluorochromes, isotypes, clones, and manufacturers.

Abbreviations: APC, allophycocyanin; FITC, fluorescein isothiocyanate; PE, phycoerythrin; PerCP/Cy5.5, peridinin–chlorophyll protein–Cyanine 5.5 tandem.

^a^Sony Biotechnology Inc., San Jose, CA, USA.

^b^Miltenyi Biotec GmbH, Bergisch Gladbach, Germany.

### 2.6. Co‐Culture Experiments With Isolated γδT Cells

The human choriocarcinoma cell line JAR (human leukocyte antigen [HLA class] I‐deficient; male; trophoblast‐derived; RRID:CVCL_0360) was used for in vitro modeling of trophoblast–immune cell interactions, in which magnetically isolated peripheral blood γδT cells were co‐incubated with JAR cells as described previously [27]. The JAR cell line was originally obtained as a generous gift from Philippe Le Bouteiller (INSERM UMR 1043, Toulouse, France) in approximately 1999 and has been maintained in our laboratory since then. The identity of the cell line was not re‐authenticated for the present experiments. According to publicly available cell line databases, the JAR cell line has not been reported as misidentified or contaminated. The HLA class I–deficient phenotype of the JAR cells was confirmed in our laboratory, and the cells were monitored and found to be free of mycoplasma contamination during the experiments. JAR cells were maintained in RPMI1640 medium (Lonza) supplemented with penicillin (1 × 10^5^ U/L) (Lonza), streptomycin (0.05 g/L) (Lonza), pyruvate (1 mM) (Gibco), and 10% FBS at 37°C in a humidified atmosphere containing 5% CO_2_. For co‐culture experiments, JAR cells were seeded in flat‐bottom 96‐well plates at a density of 2 × 10^4^ cells/well 1 day prior to peripheral blood collection and incubated overnight to reach near‐confluency. On the following day, 100 μL of the old medium was carefully aspirated and replaced with 100 μL of freshly isolated peripheral blood γδT cells (1 × 10^6^ cells/mL) resuspended in the complete culture medium. Under these conditions, co‐cultures were established at an estimated γδT:JAR effector‐to‐target ratio of approximately 2:1, selected to allow effective trophoblast–lymphocyte interaction without dominance of either cell type, in line with our previously established co‐culture model [27]. All conditions were tested in duplicate, and wells were treated with PMA (25 ng/mL) and Ionomycin (1 μg/mL) for 18 h to induce cytokine production. Following incubation, the 96‐well plates were centrifuged at 400 × *g* for 5 min, and aliquots of the supernatants were collected and stored at –80°C for subsequent cytokine analysis using cytometric bead array (CBA). Since trophoblast cells are known to produce IL‐6 [29], wells containing only JAR cells were included in all experiments. To account for any background cytokine release by JAR cells, concentrations of IL‐2, IL‐4, IL‐6, IL‐10, TNF‐α, IFN‐γ, and IL‐17A were measured in JAR cell monocultures, and these values were subtracted from the corresponding values obtained in γδT + JAR co‐cultures.

### 2.7. CBA

Cytokine profiles were assessed from co‐culture supernatants using the BD CBA, comparing γδT cells stimulated with PMA/Ionomycin alone and those co‐incubated with JAR cells under the same stimulation conditions. The BD CBA Human Th1/Th2/Th17 Cytokine Kit was used to quantify the protein levels of cytokines, including IL‐2, IL‐4, IL‐6, IL‐10, TNF‐α, IFN‐γ, and IL‐17A, in a single sample. The assay utilizes cytokine‐specific capture beads and PE‐conjugated detection antibodies, allowing simultaneous quantification of multiple cytokines by flow cytometry. Briefly, cytokine standards were prepared by serial dilution of the reconstituted standard mixture according to the manufacturer’s instructions. Individual capture bead populations were combined immediately before use to generate a mixed bead suspension. For each standard and sample, 50 μL of mixed capture beads and 50 μL of sample supernatant or cytokine standard were incubated with 50 μL of the PE detection reagent for 3 h at room temperature in the dark. Following incubation, the samples were washed with 1 mL wash buffer and centrifuged at 200 × *g* for 5 min. The supernatant was discarded, and the bead pellet was resuspended in 300 μL wash buffer prior to acquisition. Cell culture supernatants containing high cytokine levels were diluted appropriately to ensure that their fluorescence signals fell within the dynamic range of the standard curve. Data acquisition was conducted using a BD FACSCanto II flow cytometer (BD Immunocytometry Systems), with samples vortexed immediately before the measurement. Cytokine concentrations were analyzed using BD FACSDiva v6 and FCS Express v4 software based on calibration curves derived from serially diluted cytokine standards.

### 2.8. Statistics

Statistical analyses were performed using SPSS Statistics version 27.0 (IBM Corp., Armonk, NY, USA). Figures were generated using GraphPad Prism version 11 (GraphPad Software, San Diego, CA, USA). Data distribution was assessed using the Shapiro–Wilk test to evaluate normality prior to the statistical analysis. For comparisons between two independent groups, an unpaired two‐tailed Student’s *t*‐test was used when data followed a normal distribution. For non‐normally distributed data, the Mann–Whitney *U* test was applied. Differences among multiple cell populations within the same subjects were assessed using one‐way repeated‐measures ANOVA, followed by Bonferroni‐adjusted post hoc test. Comparisons between differently treated samples from the same individuals were performed using the paired *t*‐test for normally distributed data and the Wilcoxon signed‐rank test for non‐normally distributed data. Categorical variables were compared using the Fisher’s exact test. Differences were considered statistically significant at *p* ≤ 0.05.

## 3. Results

### 3.1. Baseline Distribution and Activation State of Peripheral Blood NILL Subsets in Healthy Nonpregnant and Early Pregnant Women

We performed flow cytometric analysis to evaluate the composition and the early activation state of four distinct peripheral blood NILL subsets—conventional NK cells, conventional γδT cells, and NK‐like T cell subsets (NK‐like γδT, and NK‐like non‐γδT cells)—under baseline conditions. Subset identification was based on surface expression of CD45, CD3, CD56, and TCRγδ, as detailed in the gating strategy, and activation status was determined by detecting CD69 expression, an early activation‐associated marker (Supporting Information 1: Figure S1). Notably, αβTCR expression was not directly assessed; therefore, the CD3^+^/CD56^+^/TCRγδ^−^ population is defined throughout the manuscript as NK‐like non‐γδT cells based on the phenotypic marker profile applied in this study.

Within the NILL compartment, CD3^−^ NK cells represented the predominant population in both women’s cohorts. Among CD3^+^ cell subsets, similar proportions were observed in nonpregnant women, although analysis of NK‐like T‐cell subsets revealed a significantly higher frequency of NK‐like non‐γδT cells than NK‐like γδT cells in pregnant women (Figure 1A). To define the composition of NILL subsets, each subset was expressed as a percentage of the total NILL population. Conventional NK cells accounted for 64% of NILL in nonpregnant women and 58% in pregnant women. NK‐like non‐γδT cells represented the second most prevalent subset (16% and 21%, respectively). Conventional γδT cells were present in similar proportions in both groups (~14%), whereas NK‐like γδT cells constituted the smallest subset, with frequencies of 6% in nonpregnant and 7% in pregnant women (Figure 1B).

**Figure 1:**
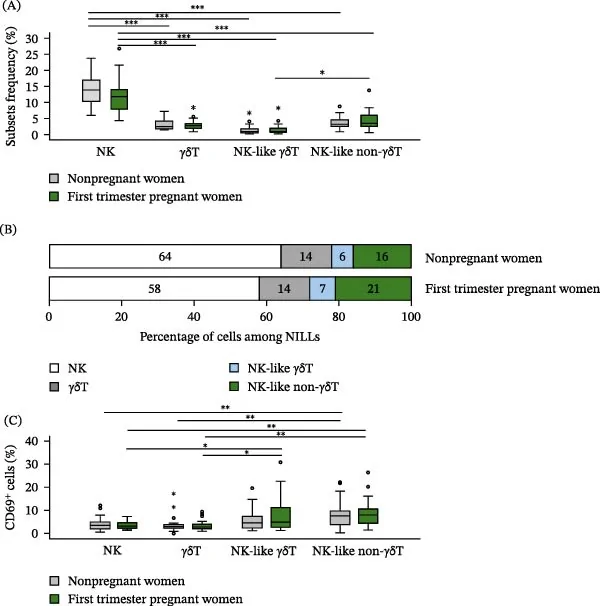
Distribution and activation state of natural and innate‐like lymphocyte (NILL) subsets in the baseline group of peripheral blood samples obtained from nonpregnant and first‐ trimester pregnant women. (A) Proportion of NILL subsets among peripheral blood mononuclear cells (PBMCs) of nonpregnant and pregnant women. Frequencies of conventional NK cells, conventional, and NK‐like γδT cells, and NK‐like non‐γδT cells are presented as percentages of total CD45^+^ PBMCs, with CD45^+^ cells defined as 100%. (B) Relative distribution of NILL subsets within the total NILL compartment of nonpregnant and pregnant women. Each subset is expressed as a proportion of the total NILL population, calculated by setting the sum of all four NILL subsets to 100%. (C) CD69 expression within NILL subsets in nonpregnant and pregnant women. Activation status is indicated by the proportion of CD69^+^ cells within each NILL subpopulation. Data are shown as mean ± SEM. Data distribution was evaluated using the Shapiro–Wilk test, and the unpaired two‐tailed Student’s *t*‐test was used for comparisons between women cohorts. For multiple comparisons among NILL subpopulations within each cohort, one‐way repeated‐measures ANOVA with Bonferroni post hoc test was applied. Sample sizes were *n* = 24 for nonpregnant and *n* = 25 for pregnant women and were consistent across all analyses. Asterisks indicate significance levels:  ^∗^ *p* ≤ 0.05,  ^∗∗^ *p* ≤ 0.01,  ^∗∗∗^ *p* ≤ 0.001.

To assess the level of recently in vivo‐activated lymphocytes, we next analyzed the surface expression of CD69, which is rapidly induced following stimulation in T cells and can also be expressed by other immune cells, including NK cells [30]. This property makes CD69 a sensitive indicator of early immune activation. As expected, CD69 expression was low in the baseline group across all subsets, consistent with freshly isolated, unstimulated peripheral blood cells. In nonpregnant samples, a significantly higher frequency of CD69^+^ cells was observed only in NK‐like non‐γδT cells compared to conventional NILL subsets. In the pregnant cohort, both NK‐like T‐cell subsets exhibited significantly higher CD69 positivity than conventional NK and γδT cells (Figure 1C).

Despite these subset‐specific findings, no statistically significant differences were observed between nonpregnant and pregnant women in the distribution or activation profiles of peripheral blood NILL populations.

Overall, these findings demonstrate a heterogeneous distribution and activation profile among peripheral blood NILL subsets, with NK cells predominating and NK‐like γδT cells representing the smallest subset, while NK‐like T‐cell subsets tended to exhibit higher baseline activation. Importantly, neither NILL subset distribution nor activation status differed significantly between nonpregnant and early pregnant women.

### 3.2. Surface Expression Intensity Profiles of ChRs on NILL Subsets Under Baseline and Antigen‐Independent Stimulation Conditions

To evaluate the migratory potential of NILL subsets based on ChR expression, we analyzed the surface expression intensity of three selected ChRs—CCR5 (a Th1‐associated receptor), CCR6 (a Th17‐associated receptor), and CXCR4 (a receptor involved in homeostatic cell migration)—by assessing MFI in peripheral blood samples from healthy nonpregnant and first‐trimester pregnant women under baseline and in vitro PMA/Ionomycin‐stimulated conditions. MFI values were used as a measure of the average receptor density per cell.

To define lineage‐specific patterns of ChR expression, we first compared ChR levels between CD3^-^ and CD3^+^ NILL cell populations. Our data revealed that conventional CD3^−^ NK cells consistently expressed all three ChRs at significantly lower intensity levels than the remaining CD3^+^ NILL subsets, independently of pregnancy or stimulation status (Figure 2A–C).

**Figure 2:**
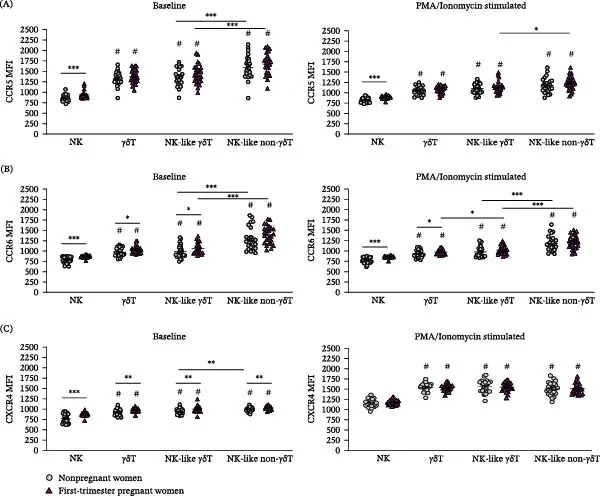
Mean fluorescence intensity (MFI) of chemokine receptors (CCR5, CCR6, and CXCR4) on peripheral blood natural and innate‐like lymphocyte (NILL) subpopulations in nonpregnant and first‐trimester healthy pregnant women under baseline and PMA/Ionomycin‐ stimulated conditions. (A) CCR5 expression intensity under baseline and PMA/Ionomycin‐stimulated conditions in peripheral NILL subpopulations in nonpregnant and first‐trimester pregnant women. (B) CCR6 expression intensity under baseline and PMA/Ionomycin‐stimulated conditions in peripheral NILL subpopulations in nonpregnant and first‐trimester pregnant women. (C) CXCR4 expression intensity under baseline and PMA/Ionomycin‐stimulated conditions in peripheral NILL subpopulations in nonpregnant and first‐trimester pregnant women. The individual data points are represented as circles (for nonpregnant women) or triangles (for first‐trimester pregnant women). Data are shown as mean ± SEM (*n* = 24 for nonpregnant and *n* = 25 for first‐trimester pregnant women). Normality of all MFI datasets was confirmed using the Shapiro–Wilk test. All data were normally distributed; therefore, parametric statistical comparisons were performed using the unpaired two‐tailed Student’s *t*‐test for comparisons between women cohorts. The comparison between baseline and stimulated samples was performed using the paired Student’s *t*‐test ( ^∗^ *p* ≤ 0.05,  ^∗∗^ *p* ≤ 0.01,  ^∗∗∗^ *p* ≤ 0.001). One‐way repeated‐measures ANOVA followed by Bonferroni post hoc test was used for multiple comparisons among NILL subpopulations within each cohort. Statistically significant differences from NK cells within the same study group (nonpregnant or first‐trimester pregnant) are marked with a hash symbol placed above the respective γδT, NK‐like γδT, or NK‐like non‐γδT data sets (^#^ *p* ≤ 0.001).

Within the CD3^+^ compartment, we next compared MFI levels between γδT cell subsets. Differences between conventional and NK‐like γδT cells were observed only under PMA/Ionomycin‐stimulated conditions, where NK‐like γδT cells exhibited significantly higher CCR6 expression intensity than conventional γδT cells in pregnant women, whereas no significant differences were observed in the nonpregnant cohort (Figure 2A–C).

To further investigate migratory differences among CD3^+^ NILL subpopulations, we compared MFI values between the two NK‐like T‐cell subsets. Under baseline conditions, NK‐like non‐γδT cells exhibited significantly higher CCR5 and CCR6 expression intensities than NK‐like γδT cells in both cohorts. In addition, this population showed higher CXCR4 expression intensity in baseline samples from nonpregnant women only. Following PMA/onomycin stimulation, this pattern was restricted to CCR6 in both cohorts and for CCR5 in the pregnant cohort only (Figure 2A–C).

We then compared ChR expression intensity between baseline and PMA/Ionomycin‐stimulated samples to assess receptor dominance patterns under different stimulation conditions. CCR5 MFI levels were consistently significantly lower in PMA/Ionomycin‐stimulated samples across all NILL subpopulations (NK, γδT, NK‐like γδT, and NK‐like non‐γδT cells) (*p* ≤ 0.01, *p* ≤ 0.001, *p* ≤ 0.001, and *p* ≤ 0.001, respectively) (Figure 2A). Similarly, most NILL subpopulations exhibited a slightly but significantly lower CCR6 receptor expression intensity in stimulated cohorts of both groups of women (*p* ≤ 0.001), except for conventional NK and NK‐like γδT cells in nonpregnant individuals (Figure 2B). In contrast, CXCR4 exhibited the highest expression intensity in PMA/Ionomycin‐stimulated samples across all NILL subpopulations, irrespective of pregnancy status (*p* ≤ 0.001) (Figure 2C).

Finally, we compared samples from nonpregnant and pregnant women to assess whether pregnancy modulates the migratory potential of maternal peripheral immune cells. Overall, MFI values showed slight but significant differences between the two cohorts. Among NILL subsets, only conventional NK cells exhibited higher CCR5 expression intensity during pregnancy, as observed under both baseline and stimulated conditions (Figure 2A). CCR6 expression intensity was higher during pregnancy under baseline conditions in conventional NK cells and both γδT cell subsets, whereas under PMA/Ionomycin stimulation, this difference was restricted to conventional NK and conventional γδT cells (Figure 2B). CXCR4 showed a distinct pattern, with all NILL subpopulations exhibiting higher expression intensity during pregnancy under baseline conditions; however, these differences were no longer detectable following stimulation (Figure 2C).

Collectively, our data reveal distinct lineage‐ and activation‐associated ChR expression intensity profiles among peripheral blood NILL subsets, with conventional NK cells exhibiting consistently lower receptor levels than those of CD3^+^ NILL populations. Notably, PMA/Ionomycin‐stimulated samples were characterized by lower CCR5 and CCR6 expression intensities but higher CXCR4 expression intensity across most NILL subsets compared with baseline samples, while pregnancy‐associated differences were generally modest and receptor‐ and subset‐specific.

### 3.3. Frequency of ChR‐Positive Cells Among NILL Subpopulations Under Baseline and Antigen‐Independent Stimulation Conditions

In this section, we analyzed the distribution of ChR^+^ cells across NILL subpopulations and compared their patterns under baseline and short‐term antigen‐independent stimulation.

Consistent with the previously observed MFI patterns, all CD3^+^ NILL subpopulations exhibited significantly higher proportions of ChR^+^ cells than CD3^-^ NK cells under all conditions in both cohorts (Figure 3A–C). Among γδT cell subsets, the proportions of ChR^+^ cells did not differ significantly between the two cell subsets in both nonpregnant and pregnant women, irrespective of activation status; notably, approximately half of γδT cells were CCR5^+^ under baseline conditions (Figure 3A–C). Differences between NK‐like T cell subsets were observed for CCR5 and CCR6, with NK‐like non‐γδT cells exhibiting higher proportions of CCR5^+^ and CCR6^+^ cells than NK‐like γδT cells across baseline and stimulated conditions in both cohorts, except for CCR5 in PMA/Ionomycin‐stimulated samples from nonpregnant women, where the difference did not reach statistical significance despite a similar trend (Figure 3A,B). In contrast, CXCR4^+^ cell proportions were comparable between NK‐like T subsets under all conditions.

**Figure 3:**
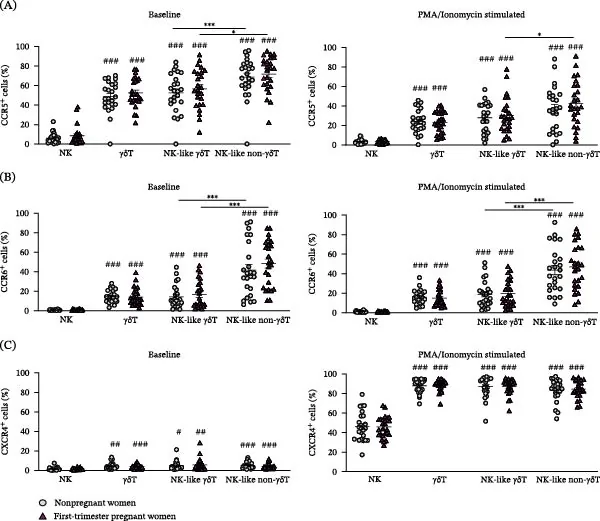
Frequency of chemokine receptor‐positive cells (CCR5, CCR6, and CXCR4) among peripheral blood natural and innate‐like lymphocyte (NILL) subpopulations in nonpregnant and first‐trimester healthy pregnant women under baseline and PMA/Ionomycin‐stimulated conditions. (A) Proportions of CCR5^+^ cells across NILL subsets under baseline and PMA/Ionomycin‐stimulated conditions. (B) Proportions of CCR6^+^ cells across NILL subsets under baseline and PMA/Ionomycin‐stimulated conditions. (C) Proportions of CXCR4^+^ cells across NILL subsets under baseline and PMA/Ionomycin‐stimulated conditions. The individual data points are represented as circles (for nonpregnant women) or triangles (for first‐trimester pregnant women). Data are shown as mean ± SEM. Each experiment was conducted with *n* = 24 for nonpregnant women and *n* = 25 for first‐trimester pregnant women. Normality was evaluated using the Shapiro–Wilk test. Statistical comparisons between women cohorts were performed using the unpaired two‐tailed Student’s *t*‐test or Mann–Whitney *U* test, depending on data distribution. Paired comparisons between baseline and stimulated conditions were assessed using the paired Student’s *t*‐test or Wilcoxon signed‐rank test, as appropriate ( ^∗^ *p* ≤ 0.05,  ^∗∗^ *p* ≤ 0.01,  ^∗∗∗^ *p* ≤ 0.001). One‐way repeated‐measures ANOVA followed by Bonferroni post hoc test was used for multiple comparisons among NILL subpopulations within each cohort. Statistically significant differences from NK cells within the same study group (nonpregnant or first‐trimester pregnant) are denoted by hash symbols placed above the respective γδT, NK‐like γδT or NK‐like non‐γδT data sets (^#^ *p* ≤ 0.05, ^##^ *p* ≤ 0.01, ^###^ *p* ≤ 0.001).

We further compared the proportions of ChR^+^ cells under baseline and PMA/Ionomycin‐stimulated conditions in both nonpregnant and pregnant cohorts to assess differences in receptor dominance between resting and in vitro‐stimulated samples. Under stimulated conditions, the proportion of CCR5^+^ cells was significantly lower across all NILL subpopulations in nonpregnant women (NK, γδT, NK‐like γδT, and NK‐like non‐γδT cells; *p* ≤ 0.05, *p* ≤ 0.001, *p* ≤ 0.001, and *p* ≤ 0.001, respectively) and similarly lower across all subsets in pregnant women (*p* ≤ 0.001 for all) (Figure 3A). In contrast, CXCR4^+^ cell proportions were significantly higher under stimulated conditions in both cohorts (*p* ≤ 0.001), with all CD3^+^ NILL subsets reaching frequencies close to or exceeding 80% (Figure 3C). Compared with the marked CCR5 and CXCR4 patterns, CCR6‐related differences were modest: under stimulated conditions, only slightly higher CCR6^+^ cell proportions were observed in conventional NK, conventional γδT and NK‐like γδT cells in nonpregnant women (*p* ≤ 0.001, *p* ≤ 0.01, and *p* ≤ 0.001, respectively), whereas during pregnancy, this difference was limited to the NK‐like γδT cell subset (*p* ≤ 0.01) (Figure 3B).

Despite slight pregnancy‐associated differences in MFI values described above, overall frequencies of ChR^+^ NILL subpopulations were comparable between the two cohorts of women (Figure 3).

Consistent with the MFI results described above, these findings further demonstrate distinct ChR expression patterns between CD3^+^ and CD3^−^ NILL subsets, while CD56^−^ and CD56^+^ γδT cell subsets showed similar frequencies of CCR5‐, CCR6‐, and CXCR4‐positive cells. Notably, PMA/Ionomycin‐stimulated samples were characterized by lower proportions of CCR5^+^ cells and markedly higher proportions of CXCR4^+^ cells than baseline samples, whereas CCR6‐related differences were modest. Importantly, the overall frequencies of ChR‐positive NILL subsets were highly comparable between nonpregnant and pregnant women.

### 3.4. ChR Co‐Expression Patterns in NILL Under Baseline and Antigen‐Independent Stimulation Conditions

To extend single‐marker analyses and identify phenotypically distinct cell populations, we assessed ChR co‐expression patterns, including dual (CCR5^+^/CCR6^+^, CXCR4^+^/CCR6^+^, and CXCR4^+^/CCR5^+^) and triple‐positive combinations under baseline and PMA/Ionomycin‐stimulated conditions, which may define lymphocyte subsets with enhanced sensitivity to multiple chemokine gradients.

Across all NILL populations, ChR co‐expression patterns largely reflected the previously observed single‐receptor and MFI results. CD3^−^ NK cells consistently exhibited the lowest frequencies of both dual‐ and triple‐positive cells, except for the CXCR4^+^/CCR5^+^ phenotype under baseline conditions in the nonpregnant cohort, where no difference was observed between NK cells and either of the two γδT‐cell subsets (Figure 4A–D).

**Figure 4:**
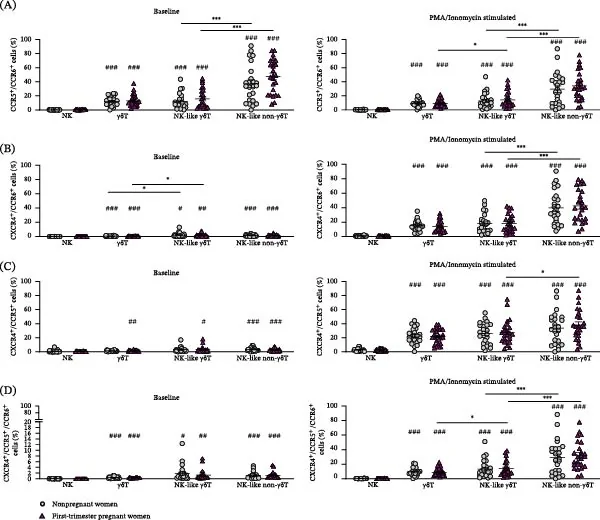
Chemokine receptor co‐expression on peripheral natural and innate‐like lymphocyte (NILL) subsets in nonpregnant and first‐trimester healthy pregnant women under baseline and PMA/Ionomycin‐stimulated conditions. Frequencies of (A) CCR5^+^/CCR6^+^, (B) CXCR4^+^/CCR6^+^, (C) CXCR4^+^/CCR5^+^, and (D) CXCR4^+^/CCR6^+^/CCR5^+^ triple‐positive cells among peripheral NILL subsets in baseline and PMA/Ionomycin‐stimulated samples. The individual data points are represented as circles (nonpregnant women, *n* = 24) or triangles (first‐trimester pregnant women, *n* = 25). Data are shown as mean ± SEM. Normal distribution was evaluated using the Shapiro–Wilk test, and statistical comparisons between women cohorts were performed using the unpaired two‐tailed Student’s *t*‐test or Mann–Whitney *U* test, depending on data normality. Paired comparisons between baseline and stimulated conditions were conducted using the paired Student’s *t*‐test or Wilcoxon signed‐rank test, depending on data distribution ( ^∗^ *p* ≤ 0.05,  ^∗∗^ *p* ≤ 0.01,  ^∗∗∗^ *p* ≤ 0.001). One‐way repeated‐measures ANOVA followed by Bonferroni post hoc test was used for multiple comparisons among NILL subpopulations within each cohort. Statistically significant differences from NK cells within the same study group (nonpregnant or first‐trimester pregnant) are denoted by hash symbols placed above the respective γδT, NK‐like γδT, or NK‐like non‐γδT data sets (^#^ *p* ≤ 0.05, ^##^ *p* ≤ 0.01, ^###^ *p* ≤ 0.001).

Interestingly, while single ChR expression was overall comparable between conventional γδT and NK‐like γδT cells, significant differences were observed at the level of double and triple co‐expression patterns, predominantly involving CCR6‐containing combinations. Under baseline conditions, NK‐like γδT cells displayed higher frequencies of CXCR4^+^/CCR6^+^ cells than conventional γδT cells in both nonpregnant and pregnant women (Figure 4B). A similar pattern was observed for CCR5^+^/CCR6^+^ and triple‐positive cells following PMA/Ionomycin stimulation, but only in the pregnant cohort (Figure 4A,D).

Analysis of NK‐like T‐cell subsets revealed distinct ChR co‐expression patterns under baseline and stimulated conditions. Under baseline conditions, co‐expression significant differences between NK‐like non‐γδT and NK‐like γδT cells were restricted to the CCR5/CCR6 ChR‐combination, with higher proportions detected in NK‐like non‐γδT cells in both nonpregnant and pregnant women (Figure 4A). This observation is in line with the higher single‐positive CCR5 and CCR6 expression previously identified in this population under resting conditions. In contrast, under PMA/Ionomycin‐stimulated conditions, differences in ChR co‐expression between the two NK‐like T cell populations became largely consistent across combinations. NK‐like non‐γδT cells exhibited higher frequencies of all dual‐ and triple‐positive ChR combinations compared to NK‐like γδT cells in both cohorts, with the sole exception of CXCR4/CCR5 co‐expression in the nonpregnant cohort, where the difference did not reach statistical significance despite a similar trend (Figure 4).

Comparison of baseline and PMA/Ionomycin‐treated samples showed that CXCR4‐containing co‐expression patterns were more frequent in stimulated samples across all NILL subpopulations in both cohorts (*p* ≤ 0.001). By contrast, lower CCR5/CCR6 co‐expression frequencies were observed in stimulated samples of conventional γδT and NK‐like non‐γδT cell subsets in both nonpregnant (*p* ≤ 0.001 and *p* ≤ 0.01, respectively) and pregnant cohorts (*p* ≤ 0.01 and *p* ≤ 0.001, respectively) (Figure 4).

Consistent with single‐receptor analyses, ChR co‐expression patterns were largely similar between nonpregnant and pregnant cohorts.

Taken together, ChR co‐expression analysis revealed slight differences between conventional and NK‐like γδT cells that were not apparent from single‐receptor expression alone, particularly in CCR6‐expressing combinations during pregnancy. Compared to baseline, PMA/Ionomycin‐stimulated samples were characterized by markedly higher frequencies of CXCR4‐containing co‐expression patterns across NILL subsets, while co‐expression profiles remained largely comparable between nonpregnant and pregnant women.

### 3.5. Expression of ChRs on Resting (CD69^−^) and Recently Activated (CD69^+^) Conventional Peripheral Blood γδT Cells in Healthy Early Pregnancy

Among NILL populations, conventional γδT cells represent a particularly relevant subset due to their unique immunological characteristics and the limited understanding of their functional role during pregnancy—especially when compared with the extensively studied NK cell population. In this part of our study, we compared ChR expression on resting (CD69^−^) and recently activated (CD69^+^) conventional γδT cells from healthy nonpregnant and first‐trimester pregnant women to assess whether early physiological activation is already associated with changes in ChR distribution patterns. Using the gating strategy shown in Supporting Information 1: Figure S1, we identified CD69^−^ resting and CD69^+^ physiologically activated γδT cells directly ex vivo and analyzed both the proportions of CCR5‐, CCR6‐, and CXCR4‐expressing cells and the corresponding MFI levels within these subsets.

Consistent with baseline single‐receptor analyses, CCR5 was the most prominently expressed ChR on γδT cells. High CCR5 expression was observed in both CD69^−^ and CD69^+^ subsets, with no significant differences between the activation states in either cohort (Figure 5A).

**Figure 5:**
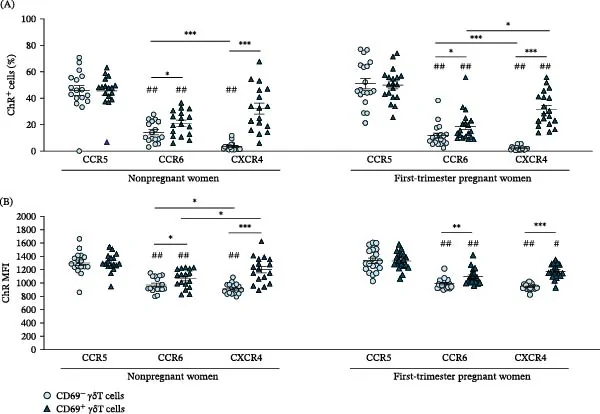
Chemokine receptor expression patterns on peripheral CD69^−^ and CD69^+^ conventional γδT cells. (A) Frequencies of CCR5‐, CCR6‐, and CXCR4‐expressing cells within CD69^−^ and CD69^+^ conventional γδT cell populations. ChR refers to chemokine receptor. (B) Median fluorescence intensity (MFI) of CCR5, CCR6, and CXCR4 on CD69^−^ and CD69^+^ conventional γδT cells. The individual data points are represented as circles (within CD69^‒^ cell gate) or triangles (within CD69^+^ cell gate). Data are shown as mean ± SEM. Data are based on analyses of samples from nonpregnant (*n* = 17) and pregnant women (*n* = 19). Normality was evaluated using the Shapiro–Wilk test, and statistical comparisons between women cohorts were performed using the unpaired two‐tailed Student’s *t*‐test ( ^∗^ *p* ≤ 0.05,  ^∗∗^ *p* ≤ 0.01,  ^∗∗∗^ *p* ≤ 0.001). One‐way repeated‐measures ANOVA followed by Bonferroni post hoc test was used for multiple comparisons among ChR‐expressing γδT‐cell populations within each CD69^−^ or CD69^+^ subset. Statistically significant differences from CCR5 data sets within the same CD69^−^ or CD69^+^ γδT‐cell subset are denoted by hash symbols placed above the respective CCR6 or CXCR4 data sets, where all comparisons were made within the same study group (nonpregnant or first‐trimester pregnant) (^#^ *p* ≤ 0.01, ^##^ *p* ≤ 0.001).

In contrast, CCR6 and CXCR4 expression were preferentially associated with the activated γδT cell compartment. Within resting CD69^−^ γδT cells, CCR6‐expressing cells were more abundant than CXCR4‐expressing cells in both cohorts, whereas in the CD69^+^, activated γδT subset, this pattern was reversed, particularly during pregnancy (Figure 5A). MFI analysis showed a similar pattern only in the nonpregnant cohort. (Figure 5B).

Proportions of ChR–positive CD69^−^ and CD69^+^ γδT cells did not differ between nonpregnant and pregnant cohorts, whereas CXCR4 MFI was slightly but significantly higher in CD69^−^ γδT cells from pregnant women (*p* ≤ 0.05) (Figure 5B).

These results indicate that CCR5 is preferentially expressed on conventional γδT cells irrespective of their in vivo activation state. In contrast, the frequency of CCR6‐ and CXCR4‐expressing γδT cells showed activation‐state‐associated expression patterns, with CXCR4 predominating over CCR6 among CD69^+^ γδT cells, although this difference reached statistical significance only in pregnant women.

### 3.6. Cytokine Production by Peripheral Conventional γδT Cells and Its Modulation by Trophoblast JAR Cells

Cytokine production by peripheral conventional γδT cells was assessed in healthy nonpregnant and pregnant women using a CBA, targeting a panel of cytokines encompassing pro‐inflammatory, anti‐inflammatory, and effector‐associated responses, including IL‐2, IL‐4, IL‐6, IL‐10, TNF‐α, IFN‐γ, and IL‐17A. To evaluate whether trophoblast‐derived signals modulate maternal immune cell cytokine responses, γδT cells were also co‐cultured with JAR cells. All cytokine concentrations shown in Figure 6 represent background‐corrected values. Importantly, except for IL‐6, JAR cells showed negligible cytokine production in monoculture under the applied conditions, supporting the interpretation that the cytokine changes observed in co‐cultures predominantly reflect γδT cell–derived responses. Mean cytokine concentrations produced by JAR cells in monoculture are summarized in the Supporting Information 2: Table S1.

**Figure 6:**
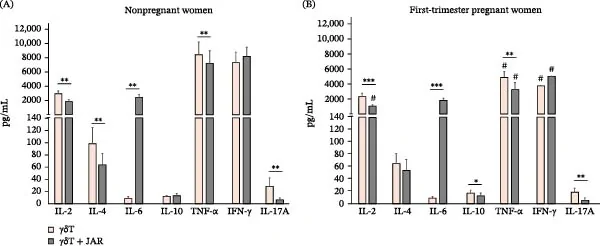
Cytokine secretion by peripheral conventional γδT cells cultured alone and co‐cultured with trophoblast JAR cells. (A) Cytokine profiles of conventional γδT cells isolated from healthy nonpregnant women (*n* = 13). (B) Cytokine profiles of conventional γδT cells isolated from healthy first‐trimester pregnant women (*n* = 14). Data are shown as mean ± SEM. Normality was tested using the Shapiro–Wilk test, and statistical comparisons between control and JAR co‐cultured conditions within each group were performed using paired Student’s *t*‐tests for normally distributed data and Wilcoxon signed‐rank tests for non‐normally distributed data ( ^∗^ *p* ≤ 0.05,  ^∗∗^ *p* ≤ 0.01,  ^∗∗∗^ *p* ≤ 0.001). Comparisons between nonpregnant and pregnant women were conducted using the unpaired two‐tailed Student’s *t*‐tests or Mann–Whitney *U* tests, as appropriate. Statistically significant differences between the nonpregnant and pregnant groups are marked with hash symbols placed above the bars corresponding to the pregnant group (^#^ *p* ≤ 0.05).

Under both basal and co‐culture conditions, IL‐2, TNF‐α, and IFN‐γ were the most abundantly produced cytokines, showing comparable profiles across the nonpregnant and pregnant groups. Co‐culture with JAR cells significantly modulated γδT cell cytokine secretion. Statistically significant changes included increased IL‐6 production, along with reduced levels of IL‐2, IL‐17A, and TNF‐α in both groups. IL‐4 secretion was significantly suppressed only in nonpregnant women, while IL‐10 production was significantly lower exclusively in the pregnant group. Although IFN‐γ levels increased following co‐culture, this change did not reach statistical significance in any of the studied groups; however, a trend toward significance was observed in pregnant samples (*p* = 0.0538) (Figure 6A,B).

To evaluate whether pregnancy modulates the cytokine production profile of γδT cells, we next compared the production levels between pregnant and nonpregnant women under both monoculture and co‐culture conditions. γδT cells from pregnant women under monoculture conditions produced significantly lower levels of the inflammatory cytokines TNF‐α and IFN‐γ compared with nonpregnant controls; however, following co‐culture with JAR cells, IL‐2 production was also slightly but significantly reduced, while the significant suppression of TNF‐α and IFN‐γ levels compared with nonpregnant controls was preserved (Figure 6A,B).

Taken together, circulating γδT cells exhibited a predominantly pro‐inflammatory cytokine profile characterized by high IL‐2, TNF‐α, and IFN‐γ production, which is modulated by trophoblast cells. Pregnancy was associated with lower production of the pro‐inflammatory cytokines TNF‐α and IFN‐γ, indicating a less inflammatory cytokine profile of peripheral γδT cell.

## 4. Discussion

Early human pregnancy is characterized by the accumulation of maternal innate‐like immune cells at the feto‐maternal interface, where NK cells represent the dominant population. Previous studies have demonstrated that the phenotype and function of these cells are tightly regulated to support maternal immune tolerance towards the developing fetus [31]. While the origin and adaptation of decidual NK cells have been extensively studied, considerably less is known about the recruitment and local tissue‐specific shaping of decidual TCRγδ^+^ T‐cell subsets. Chemokine‐mediated migration represents a plausible mechanism contributing to their accumulation within the decidua. In early pregnancy, trophoblast cells produce a variety of chemokines, including CXCL12 [32] and CXCL16 [17], whose corresponding receptors are expressed on several maternal immune cell populations and may guide their migration toward the pregnant uterus. In this context, characterizing ChR profiles in peripheral blood, which is more accessible to clinicians, may complement existing knowledge and provide further insight into the migratory potential and regulation of maternal innate‐like immune cells during the first trimester. This approach could also facilitate future investigations of possible alterations in pathological settings, including the increased expression of inflammatory ChRs.

In this study, we examined the expression profiles of three selected ChRs—CCR5, CCR6, and CXCR4—across peripheral blood NILL subpopulations, whose respective ligands (CCL3/CCL4/CCL5, CCL20, and CXCL12) have been detected in placental and decidual tissues [33–35]. To explore activation‐dependent regulation, ChR expression was additionally analyzed following short‐term, antigen‐independent stimulation, enabling a standardized comparison across NILL subsets. In a subsequent step, we focused on γδT cells to characterize their functional cytokine responses in a distinct experimental setting. Here, γδT cells were analyzed following PMA/Ionomycin stimulation alone or in co‐culture with trophoblast‐like JAR cells, allowing the assessment of trophoblast‐associated modulation of γδT‐cell cytokine responses.

When investigating NILL populations, we found that CD3^−^ and CD3^+^ NILL subsets display distinct ChR expression profiles. Conventional CD3^−^ NK cells exhibited markedly lower frequencies of ChR^+^ cells than CD3^+^ NILL subsets, a finding consistently supported by both cell frequency analyses—including single‐, double‐, and triple ChR co‐expression—and MFI data. These results indicate a preferential association of CCR5, CCR6, and CXCR4 with CD3^+^ NILL populations, suggesting a greater potential for using these receptors for peripheral migration. This observation is consistent with previous reports showing low CCR5 and CCR6 expression on NK cells [36] and preserved uterine NK cell accumulation during pregnancy in CCR5‐deficient mouse models [37]. Taken together, our data suggest that, in contrast to CD3^+^ NILL subpopulations, conventional NK cells may be less dependent on CCR5‐ and CCR6‐mediated migration and may rely on alternative mechanisms to regulate their trafficking.

Given their innate‐like, MHC‐independent recognition and enrichment at mucosal barriers, TCRγδ^+^ lymphocytes represent a relevant but understudied population during pregnancy. Here, we found that approximately half of these cells were CCR5‐positive in both nonpregnant and pregnant cohorts, exhibiting pronounced receptor expression intensity. Similarly, previous flow cytometry studies have shown that approximately 60% of peripheral γδT cells express CCR5, representing a substantial fraction of the circulating γδT cell pool [38]. As CCR5^+^ γδT cells are associated with an effector phenotype and exhibit pro‐inflammatory activity, including elevated IFN‐γ production under inflammatory conditions [39], our findings further support the established role of γδT cells in inflammatory responses [40]. The persistence of this inflammatory characteristic during early pregnancy suggests that peripheral TCRγδ^+^ cells maintain their functional identity. In contrast, surface CXCR4 expression was uniformly low across both γδT cell subsets, reaching only about one‐tenth of the CCR5 level (~5%). According to previous reports, the localization of CXCR4 in lymphocytes is influenced by factors such as cell isolation and culture duration. Low surface levels were reported on freshly isolated lymphocytes, while much higher proportions were detected by intracellular staining. In addition, a simple 1‐h culture at 37°C increased the surface expression of the CXCR4 receptor, and longer incubation resulted in an even higher expression, suggesting mobilization from an intracellular pool [41, 42]. These observations could explain our finding of a very low percentage of surface CXCR4^+^ cells in freshly isolated, nonstimulated lymphocytes. On the other hand, the investigation of CCR6, a ChR known to be expressed on IL‐17‐producing γδT cells [43], revealed modest expression levels in the baseline group of TCRγδ^+^ cells. This modest level was also found by Brandes et al. [38], who reported that the ratio of CCR6‐expressing γδT cells was 20%. Nonetheless, this receptor was found to play a role in directing the migration of regulatory T cells during human pregnancy through the interaction with its ligand CCL20 [23] and is required for the recruitment of IL‐17‐ and IL‐22‐producing γδT cells into injured tissues [44]. Although the proportion of CCR6‐expressing peripheral maternal γδT cells appears moderate, it may still guide the migration of minor γδT cell subsets with regulatory or IL‐17‐producing potential toward inflammatory sites, such as the feto‐maternal interface during early pregnancy. In addition, examining ChR co‐expression patterns revealed, as expected, a very small fraction of TCRγδ^+^ cells co‐expressing CXCR4. Notably, the proportion of CCR5^+^/CCR6^+^ γδT cells was comparable to the overall frequency of CCR6^+^ γδT cells, indicating that nearly all CCR6^+^ cells simultaneously express CCR5 as well. Such dual co‐expression may enhance their sensitivity to a broader range of inflammatory chemokines, increasing their capacity to respond to diverse migratory signals in complex immune environments.

Leukocyte migration is a complex process in which ameboid motility is commonly employed; however, cells can shift between motility types depending on environmental factors—a phenomenon known as migratory plasticity [45]. This adaptability is also reflected at the level of ChR expression, which can change following different stimulatory signals. The direction of CXCR4 modulation after PMA/Ionomycin stimulation remains controversial in the literature. While several studies report receptor downregulation [46, 47], others describe upregulation [48], suggesting that outcomes are highly dependent on the experimental design and cell type. In our experiment, we observed different ChR dominance and expression profiles between resting and stimulated cells. Regarding the proportion of ChR‐expressing NILL subpopulations following PMA/Ionomycin treatment, CXCR4 was the most frequently detected receptor. These findings suggest that CXCR4 may serve as the dominant ChR under these conditions, whereas CCR5 remains more prevalent in unstimulated cells. Importantly, this pattern was preserved during pregnancy, suggesting that activation‐associated regulation of CXCR4 and CCR5 is maintained during pregnancy. Such rapid migratory reprogramming upon in vitro TCR triggering was reported in a prior study, in which resting cells exhibited a pro‐inflammatory ChR repertoire, whereas activated cells upregulated homing ChRs and downregulated inflammatory receptors such as CCR5 and CCR2 [38]. Thus, our results support the hypothesis that resting lymphocytes may have distinct migratory potentials that change upon encountering a stimulus, suggesting different functional potentials depending on environmental signals. Across both examined TCRγδ^+^ subsets, the CCR6 expression pattern observed after stimulation showed only occasional subtle differences compared with the baseline profile, which, although sometimes reaching statistical significance, were unlikely to be biologically relevant. Unlike CCR5, which showed a surprisingly low expression in stimulated samples, CCR6 showed no clear change in expression following stimulation.

A further key aspect of this study was to determine whether physiological activation affects ChR expression patterns. We compared resting and physiologically activated conventional γδT cells using CD69 to identify the activated subset among unstimulated cells. CD69 was selected because it is rapidly and transiently induced upon stimulation. Beyond serving as an early activation marker, CD69 is functionally linked to lymphocyte trafficking through the downregulation of sphingosine‐1‐phosphate receptor 1 (S1PR1). S1PR1 normally promotes lymphocyte egress along sphingosine‐1‐phosphate (S1P) gradients, while its downregulation limits S1P‐dependent cell migration [49]. Thus, the CD69^+^ phenotype identifies a recently activated state that is also associated with the altered regulation of lymphocyte trafficking, providing a relevant context to examine whether activation is accompanied by changes in ChR expression. Notably, CCR5 expression levels were lower in PMA/Ionomycin‐stimulated samples, whereas physiological activation was not associated with changes in its levels but was accompanied by higher CCR6 expression. Interestingly, CXCR4 expression patterns were prominent in both physiologically activated and PMA/Ionomycin‐stimulated settings, although the proportion of CXCR4‐positive cells was substantially higher following in vitro stimulation. Moreover, under both activation conditions, CXCR4 was higher than CCR6, while without activation, CCR6 dominated. Importantly, because CD69 detection alone does not reveal the nature of the activating stimulus and given that our results differed from those obtained under in vitro stimulation, it is evident that the type of activation profoundly influences ChR regulation. This is in line with available data showing that distinct stimuli are capable of influencing ChR expression [48, 50]. This complexity suggests that drawing meaningful conclusions about ChR dynamics requires examining multiple activation contexts.

γδT cells can be divided into distinct subsets based on various characteristics, and one of the most intriguing classification markers is CD56 co‐expression. In mice, CD56^+^ γδT cells exhibit a memory‐cell phenotype, express several NK cell receptors, and produce high levels of IFN‐γ [51]. In humans, this population remains largely underinvestigated, although about half of peripheral blood CD56^+^ T cells are γδT cells [25]. Our previous work demonstrated that NK‐like γδT cells have remarkable cytotoxic potential, and the frequency of CD56‐bearing cells among γδT lymphocytes increases in peripheral blood during the second half of healthy human pregnancy [25]. In the present study, we compared conventional and NK‐like γδT cells, and we found that, while the frequencies of ChR single‐positive cells were similar, NK‐like γδT cells showed a slightly higher tendency for certain ChR co‐expression, particularly during pregnancy. Although these differences were subtle, they suggest that conventional and NK‐like γδT cells may differ in their ChR profiles, supporting further investigations using a broader range of ChRs.

Although conventional γδT cells are well recognized for their cytotoxic effector functions, they can also shape immune responses through cytokine production—predominantly with proinflammatory and regulatory properties [52]. We found that peripheral conventional γδT cells can produce all tested cytokines in different ratios; however, IL‐2, TNF‐α, and IFN‐γ appeared to be the most relevant in both nonpregnant and pregnant women. This suggests that this cell population maintains its broad cytokine‐producing capacity during first‐trimester pregnancy. We further examined whether pregnancy, which requires tight regulation of potentially harmful immune activity, modulates their cytokine profile. We found that the production of proinflammatory cytokines, TNF‐α and IFN‐γ, was significantly reduced during pregnancy. Rather than reflecting a loss of effector capacity, this finding is consistent with controlled attenuation of proinflammatory potential, which may help prevent excessive immune activation while preserving immune responsiveness during early gestation.

Moreover, by the late first to early second trimester of human pregnancy, the hemochorial placental circulation becomes fully established, with maternal blood continuously perfusing the intervillous space. Consequently, maternal peripheral blood lymphocytes can come into direct contact with trophoblast cells covering the chorionic villi [53]. This anatomical arrangement provides an opportunity for trophoblast‐derived signals to modulate circulating maternal immune cells. Additionally, vesicles shed from the syncytiotrophoblast can enter the maternal circulation, providing further opportunities for modulating maternal immune cell function [54]. We examined how this interaction affects the cytokine‐producing capacity of conventional γδT cells and found a coordinated modulation characterized by downregulation of the proinflammatory cytokines IL‐2, TNF‐α, and IL‐17A, alongside a marked upregulation of IL‐6. Overall, our results suggest that trophoblast‐derived signals modulate the cytokine profile of peripheral γδT cells and attenuate the production of several proinflammatory cytokines, which could be deleterious to the developing embryo. Although it was produced at low levels, the observed reduction of IL‐17A upon co‐culture with trophoblast cells may still contribute to maintaining the immune balance during early pregnancy. This is supported by previous studies on idiopathic recurrent pregnancy loss, where IL‐17 production by peripheral CD4^−^/CD8^−^ double‐negative γδT cells was elevated compared to healthy pregnant women [55]. Notably, decidual γδT cells have been shown to support successful pregnancy. Their high‐level production of the anti‐inflammatory Th2 cytokine IL‐10 during the first trimester, along with its promoting effect on trophoblast proliferation and invasion, was originally described by Fan et al. [7]. Our findings show that peripheral conventional γδT cells can also produce IL‐10 during early gestation, albeit at very low levels. However, trophoblast contact further attenuated this production, indicating that peripheral conventional γδT cells retain a predominantly inflammatory profile and that robust IL‐10 production is likely restricted to the decidual compartment. By contrast, IL‐6 is a multifunctional cytokine that cannot be strictly categorized as Th1 or Th2, despite possessing several proinflammatory properties. During early gestation, IL‐6 plays a crucial role in regulating trophoblast differentiation and proliferation, and in human trophoblast cell lines, it has been shown to exert chemotactic and migration‐enhancing effects [56]. Consistent with this, we found that IL‐6 was the most abundantly secreted cytokine by trophoblast cells cultured alone, suggesting a prominent intrinsic role of trophoblast‐derived IL‐6. Moreover, the increased production of IL‐6 by γδT cells following co‐culture with trophoblasts further supports the possibility that γδT cells may contribute to establishing an appropriate immune milieu during pregnancy by responding to local trophoblast‐derived signals.

Nevertheless, cytokines produced upon cellular activation are such functional outputs that can also actively regulate ChR expression, thereby shaping migratory programs. Beyond the observed changes in ChR expression patterns on γδT cell subsets after stimulation, it is important to consider the underlying mechanisms. Because PMA/Ionomycin stimulation robustly induces cytokine production, the ChR expression patterns observed under these conditions may, at least in part, reflect the influence of the activation‐associated cytokine milieu. Supporting this, Bryant et al. [57] showed that IL‐2, IL‐6, and TNF‐α can enhance CXCR4 and integrin expression on peripheral T cells, highlighting the link between the cytokine milieu and migratory or adhesion profiles. Additionally, IL‐4 was shown to downregulate CCR5 while upregulating CXCR4 expression on peripheral CD4^+^ T cells [58]. These observations support the concept that the predominant CXCR4 expression observed in PMA/Ionomycin‐treated samples may, at least in part, reflect cytokine‐mediated effects induced by activation. Such CXCR4 dominance may contribute to enhanced migratory capacity toward SDF‐1‐rich environments, including the feto‐maternal interface. Moreover, it is well documented that TNF‐α and IFN‐γ enhance the transendothelial migration of peripheral blood T cells through multiple mechanisms [59–61], which may similarly influence the migratory potential of γδT cells. Although peripheral conventional γδT cells produce lower amounts of TNF‐α and IFN‐γ during pregnancy, these levels could remain sufficient to promote their transendothelial migration. Thus, conventional γδT cells could potentially modulate their own migratory behavior not only via ChR interactions but also via autocrine cytokine‐mediated mechanisms.

To date, several hypotheses have been suggested to explain the origin of DICs, particularly NK cells. In this regard, CXCR4^+^ decidual NK cells were examined by Tao et al. [62], who found that this population may originate from both CXCR4^−^ and CXCR4^+^ peripheral NK cells, suggesting that trophoblast‐derived signals can induce the maturation of CXCR4^−^ NK cells toward a CXCR4^+^ phenotype. Additionally, it was also reported that treating peripheral NK cells with decidual cytokines can alter their ChR profile, resulting in patterns resembling those of their decidual counterparts [15]. It is therefore plausible that pregnancy‐related alterations in ChR expression could take place on the tissue‐resident lymphocytes, particularly in the decidua, where the continuous presence of locally secreted factors may shape receptor profiles. Accordingly, while ChR‐guided migration from the periphery to the decidua is essential, pregnancy‐specific ChR profiles are presumably established under the influence of the materno‐fetal microenvironment. This could support the hypothesis that immune cells might first migrate to the decidua and subsequently undergo local ChR modulation, including the downregulation of receptors required for further trafficking, thereby promoting their retention at the feto‐maternal interface. Consequently, this may explain why we found remarkably similar ChR expression profiles between pregnant and nonpregnant peripheral blood samples, with only slight differences in receptor expression intensity. Although these differences were modest, they may still be functionally relevant given the high sensitivity of ChR–ligand interactions and the close correlation between surface receptor density and immune cell migratory responsiveness [63, 64]. However, one more plausible explanation regarding the absence of marked pregnancy‐associated differences in ChR^+^ cell ratios is that decidual lymphocytes may also derive from pre‐existing tissue‐resident populations undergoing local differentiation and phenotypic adaptation shaped by the feto‐maternal environment. Supporting this, Mincheva‐Nilsson et al. [65] proposed a model of intradecidual γδT cell development based on the identification of three γδT cell subpopulations in the decidua, hypothesized to represent distinct phases of local differentiation. Importantly, our observation of minimal differences in the frequency of ChR^+^ γδT cell subsets between the investigated peripheral blood cohorts suggests a conserved subset distribution during first‐trimester pregnancy.

Finally, some limitations of the present study also point toward relevant directions for further investigation. As pregnancy‐associated immune alterations may persist for some time after delivery, information on the time elapsed since previous pregnancies could provide important context for interpreting the immune characteristics, especially in nonpregnant controls; however, these data were not available and could therefore not be considered in our analysis. Our investigation was restricted to peripheral blood, whereas analysis of decidual samples could reveal tissue‐specific ChR patterns that are not detectable in the circulation and thereby provide insight into the local regulation of NILL trafficking at the feto‐maternal interface. Furthermore, only first‐trimester samples were examined; extending these analyses to later stages of pregnancy may identify gestational age–dependent changes, given the dynamic nature of maternal immune regulation throughout gestation. The present study also focused on a selected panel of ChRs. Considering the potentially complex receptor repertoire of individual NILL subsets, investigating a broader range of ChRs would provide a more comprehensive characterization of their migratory phenotype. In addition, ChR expression was assessed phenotypically without directly evaluating the migratory activity. Functional migration assays would therefore be valuable for determining the contribution of individual ChRs to the migration of NILL subsets, thereby clarifying the biological significance of the expression patterns observed in the present study.

## 5. Conclusions

Taken together, our findings demonstrate distinct ChR expression patterns among peripheral NILL subsets, with clear differences between CD3^+^ and CD3^−^ populations. Within the γδT‐cell compartment, both conventional and NK‐like γδT cells displayed a CCR5‐dominated ChR profile under resting conditions, while the marked differences observed following activation highlight the cellular activation state as an important determinant of ChR expression. Our study provides novel insight into the ChR profile of the highly under‐investigated NK‐like γδT‐cell subset, indicating that its phenotypic distinction from conventional γδT cells may be more sensitively reflected by specific ChR co‐expression profiles than by individual receptor expression. The investigated peripheral ChR patterns were largely preserved during first‐trimester pregnancy, suggesting that pregnancy‐associated regulation of ChR expression may occur within the local maternal–fetal environment rather than through extensive systemic reprogramming. In addition, we have shown that peripheral conventional γδT cells exhibited a broad cytokine‐producing capacity, dominated by IL‐2, TNF‐α, and IFN‐γ, which was modulated during pregnancy and in response to trophoblast‐derived signals. Together, these findings advance our understanding of the phenotypic and immunological characteristics of peripheral γδT‐cell populations and highlight the importance of further investigating their regulation and function during human pregnancy.

**Nomenclature**
CBA: Cytometric bead array
CCL: C‐C motif chemokine ligand
CCR: C‐C motif chemokine receptor
CD: Cluster of differentiation
ChR: Chemokine receptor
CXCL: C‐X‐C motif chemokine ligand
CXCR: C‐X‐C motif chemokine receptor
DICs: Decidual immune cells
FBS: Fetal bovine serum
FMO: Fluorescence minus one
FOXP3: Forkhead box P3
HLA: Human leukocyte antigen
IFN: Interferon
IL: Interleukin
mAb: monoclonal antibody
MFI: Mean fluorescence intensity
MIP‐3α: Macrophage inflammatory protein‐3α
NILL: Natural and innate‐like lymphocytes
NK: Natural killer
PBMC: Peripheral blood mononuclear cells
PBS: Phosphate‐buffered saline
PFA: Paraformaldehyde
PMA: Phorbol myristate acetate
RANTES: Regulated upon activation normal T cell expressed and secreted
S1P: Sphingosine‐1‐phosphate
S1PR1: Sphingosine‐1‐phosphate receptor‐1
SDF‐1: Stromal cell‐derived factor‐1
TCR: T‐cell receptor
Th: T helper
TNF: Tumor necrosis factor
Tregs: T regulatory cells.

## Author Contributions

**Banan Alrawashdeh:** conceptualization, investigation, methodology, data curation, formal analysis, visualization, writing – original draft. **Jasper Nörenberg**: investigation, methodology, formal analysis, writing – review. **Pál Jáksó**: investigation, methodology, resources, writing – review. **Barbara Forró and Mátyás Meggyes**: investigation, writing – review. **Ákos Várnagy**: resources, writing – review. **Aliz Barakonyi**: conceptualization, methodology, project administration, supervision, validation, funding acquisition, writing – original draft, review, and editing.

## Funding

This research was funded by the PTE ÁOK KA Grant (Grant KA‐2025‐02) and by the “National Laboratory on Human Reproduction” (Project Number RRF‐2.3.1‐21‐2022‐00012), titled National Laboratory on Human Reproduction has been implemented with the support provided by the Recovery and Resilience Facility of the European Union within the framework of Programme Széchenyi Plan Plus.

## Disclosure

All authors have read and approved the final manuscript. This study was presented as an oral communication at the European Society for Reproductive Immunology (ESRI) Conference, Leiden, October 2024, where preliminary results were shown. The corresponding abstract (https://doi.org/10.1016/j.jri.2024.104357) was published in the Journal of Reproductive Immunology, Volume 167 (February 2025), as part of the conference proceedings.

## Ethics Statement

Our study was reviewed and approved by the local Ethics Committee (5643‐PTE 2019 and 5643‐PTE 2023). It was conducted in accordance with the Declaration of Helsinki, and all participants—both pregnant and nonpregnant healthy women—provided written informed consent before participation.

## Consent

The authors have nothing to report.

## Conflicts of Interest

The authors declare no conflicts of interest.

## Supporting Information

Additional supporting information can be found online in the Supporting Information section.

## Supporting information


**Supporting Information 1** Figure S1: Stepwise flow cytometric gating strategy for identification of NILL subpopulations, including conventional NK cells and defined CD3^+^ cell subsets based on CD56, TCRγδ, and CD69 expression.

**Supporting Information 2** Table S1: Quantification of cytokine secretion (IL‐2, IL‐4, IL‐6, IL‐10, TNF‐α, IFN‐γ, and IL‐17A) by JAR cells cultured alone, determined by cytometric bead array.

## Data Availability

The data that support the findings of this study are available from the corresponding author upon reasonable request.
