# Supplementary material for: Highly Conserved but Activation‐Dependent CCR5, CCR6, and CXCR4 Chemokine Receptor Expression and Trophoblast‐Mediated Cytokine Profile of Peripheral Gamma‐Delta T Cells During Early Human Pregnancy
Source: J Immunol Res. 2026 Oct 23;2026:7743945. doi: 10.1155/jimr/7743945 (PMC13691526; doi:10.1155/jimr/7743945)
Supplement: Supplementary file 1 [file JIMR-2026-7743945-s001.docx]

Supplementary Figure S1: Gating strategy for Natural and Innate-Like Lymphocyte (NILL) subpopulations

Flow cytometry plots showing the stepwise gating strategy. Lymphocytes were first gated based on forward and side scatter (FSC/SSC), followed by the exclusion of doublets by single-cell gating. CD45^+^ cells were then gated from the single-cell population to identify leukocytes, which were subsequently divided into CD3^+^ and CD3^‒^ subsets. Within CD3^‒^ gate, only conventional NK cells were included in the analysis. The CD3⁺ gate included CD56⁻/TCRγδ⁺, CD56⁺/TCRγδ⁺, and CD56⁺/TCRγδ⁻ cell subsets. Resting (CD69⁻) and recently activated (CD69^+^) γδT cells were identified based on CD69 expression within the CD56⁻/TCRγδ⁺ gate, which defines the conventional γδT cell population.
