# Supplementary material for: Highly Conserved but Activation‐Dependent CCR5, CCR6, and CXCR4 Chemokine Receptor Expression and Trophoblast‐Mediated Cytokine Profile of Peripheral Gamma‐Delta T Cells During Early Human Pregnancy
Source: J Immunol Res. 2026 Oct 23;2026:7743945. doi: 10.1155/jimr/7743945 (PMC13691526; doi:10.1155/jimr/7743945)
Supplement: Supplementary file 2 [file JIMR-2026-7743945-s002.docx]

**Supplementary Table S1: Cytokine production by choriocarcinoma JAR cells**

Cytokine concentrations (IL-2, IL-4, IL-6, IL-10, TNF-α, IFN-γ, and IL-17A) were measured in supernatants of JAR cells cultured alone by a Cytometric Bead Array (CBA) assay. All values are reported as mean ± standard error of the mean (SEM) (n = 21) and are expressed in pg/mL. Values below the detection limit may appear negative due to curve fitting.

| **Cytokine** | **Concentration**  (pg/mL, Mean ± SEM) |
| --- | --- |
| IL-2 | *0.47 ± 0.50* |
| IL-4 | *−0.64 ± 0.67* |
| IL-6 | *311.20 ± 49.91* |
| IL-10 | *0.21 ± 0.26* |
| TNF-α | *5.77 ± 1.18* |
| IFN-γ | *0.40 ± 0.27* |
| IL-17A | *0.19 ± 0.25* |
